# Radiomics analysis of bone marrow biopsy locations in [^18^F]FDG PET/CT images for measurable residual disease assessment in multiple myeloma

**DOI:** 10.1007/s13246-023-01265-0

**Published:** 2023-05-08

**Authors:** Eva Milara, Rafael Alonso, Lena Masseing, Alexander P. Seiffert, Adolfo Gómez-Grande, Enrique J. Gómez, Joaquín Martínez-López, Patricia Sánchez-González

**Affiliations:** 1 Biomedical Engineering and Telemedicine Centre, ETSI Telecomunicación, Center for Biomedical Technology, Universidad Politécnica de Madrid, 28040 Madrid, Spain; 2 Department of Hematology and Instituto de Investigación Sanitaria (imas12), Hospital Universitario, 12 de Octubre, 28041 Madrid, Spain; 3 Clinical Research Hematology Unit, Centro Nacional de Investigaciones Oncológicas (CNIO), 28029 Madrid, Spain; 4 Centro de Investigación Biomédica en Red Cáncer (CIBERONC), Madrid, Spain; 5 Facultad de Medicina, Universidad Complutense de Madrid, 28040 Madrid, Spain; 6 Department of Nuclear Medicine, Hospital Universitario, 12 de Octubre, 28041 Madrid, Spain; 7 Centro de Investigación Biomédica en Red de Bioingeniería, Biomateriales y Nanomedicina, Instituto de Salud Carlos III, 28029 Madrid, Spain

**Keywords:** Multiple myeloma, Measurable residual disease, Radiomic features, Bone marrow, Biopsy, [^18^F]FDG PET

## Abstract

The combination of visual assessment of whole body [^18^F]FDG PET images and evaluation of bone marrow samples by Multiparameter Flow Cytometry (MFC) or Next-Generation Sequencing (NGS) is currently the most common clinical practice for the detection of Measurable Residual Disease (MRD) in Multiple Myeloma (MM) patients. In this study, radiomic features extracted from the bone marrow biopsy locations are analyzed and compared to those extracted from the whole bone marrow in order to study the representativeness of these biopsy locations in the image-based MRD assessment. Whole body [^18^F]FDG PET of 39 patients with newly diagnosed MM were included in the database, and visually evaluated by experts in nuclear medicine. A methodology for the segmentation of biopsy sites from PET images, including sternum and posterior iliac crest, and their subsequent quantification is proposed. First, starting from the bone marrow segmentation, a segmentation of the biopsy sites is performed. Then, segmentations are quantified extracting SUV metrics and radiomic features from the [^18^F]FDG PET images and are evaluated by Mann–Whitney U-tests as valuable features differentiating PET+/PET− and MFC+ /MFC− groups. Moreover, correlation between whole bone marrow and biopsy sites is studied by Spearman *ρ* rank. Classification performance of the radiomics features is evaluated applying seven machine learning algorithms. Statistical analyses reveal that some images features are significant in PET+/PET− differentiation, such as SUV_max_, Gray Level Non-Uniformity or Entropy, especially with a balanced database where 16 of the features show a *p* value < 0.001. Correlation analyses between whole bone marrow and biopsy sites results in significant and acceptable coefficients, with 11 of the variables reaching a correlation coefficient greater than 0.7, with a maximum of 0.853. Machine learning algorithms demonstrate high performances in PET+/PET− classification reaching a maximum AUC of 0.974, but not for MFC+/MFC− classification. The results demonstrate the representativeness of sample sites as well as the effectiveness of extracted features (SUV metrics and radiomic features) from the [^18^F]FDG PET images in MRD assessment in MM patients.

**Supplementary Information:**

The online version contains supplementary material available at 10.1007/s13246-023-01265-0.

## Introduction

Multiple Myeloma (MM) is a malignancy characterized by the excessive growth of monoclonal plasma cells in the bone marrow that accounts for about 10% of all hematologic cancers [1–3]. The main consequence of this overproduction is an uncontrolled production of immunoglobulins along with various symptoms including anemia, bone lesions, infections, hypercalcemia, renal failure, fatigue, and pain [4, 5]. The last decade, thanks to novel therapies, the achievement of deeper responses is becoming more likely for MM patients [6]. However, subclinical levels of tumor burden, known as Measurable Residual Disease (MRD), are still detectable using techniques with higher sensitivity as compared to conventional serum and urine protein evaluation [7]. The MRD, also called Minimal Residual Disease, has been defined by the International Myeloma Working Group (IMWG) [6] as one of the most important features for identifying patients with different survival outcomes.

Currently, the most widely used method for MRD quantification consists on a bone marrow biopsy/aspiration taken from the pelvis or sternum of the patient. Once the sample is extracted, residual tumor burden is quantified using Multiparameter Flow Cytometry (MFC) or Next-Generation Sequencing (NGS) [3, 6]. Despite the high sensitivity of these techniques, a number of MRD- patients, i.e. patients with a negative biopsy result, still experience relapse due to the small representation or poor quality of the taken sample, the heterogeneity of the bone marrow involvement, the existence of extramedullary disease or the insufficient sensitivity of the applied technique [7, 8]. For this reason, the combined use of MFC or NGS with visual evaluation of imaging by low-dose whole body CT or PET/CT with fluorine-18 fluorodeoxyglucose ([^18^F]FDG) is becoming increasingly important in the MRD detection [9, 10]. In fact, the IMWG recommends this imaging technique to evaluate the extent of bone disease and the presence of extramedullary disease [11, 12]. Despite the lack of standardization in the interpretation of PET studies, Nanni et al. [13] have proposed a reading model for response assessment, called IMPeTUs, which has been established as the standardized criterion for the visual interpretation of PET based on the Deauville Criteria [14]. However, this criterion is controversial for bone marrow evaluation in difficult-to-assess cases. Consequently, bone marrow analysis based simply on visual interpretation remains very limited.

To assess MRD by [^18^F]FDG PET imaging, not only visual evaluation is performed, but also quantification of activity concentration, especially the Standardized Uptake Value (SUV) and its maximum value (SUV_max_) [10, 15]. However, SUV values can be altered by a wide variety of artifacts. Therefore, the IMPeTUs criteria have excluded SUV as a valid MRD marker [13, 16]. In other pathologies, the quantification of [^18^F]FDG PET images have evolved to analysis based on textural features within the field of radiomics, increasing the level of quantitative information to be extracted from the image, with the aim of improving diagnostic accuracy and prognostic prediction [17–20]. Indeed, for patients newly diagnosed MM, radiomics quantification of [^18^F]FDG PET images have been studied as prognostic indicators of worse survivall [21, 22]. Moreover, machine learning (ML) models based on radiomic features for MM diagnosis [23] and MRD detection [24] with [^18^F]FDG PET images has been previously studied.

In the study of Milara et al. [24], a segmentation methodology along with the analysis of radiomic features extracted from [^18^F]FDG PET/CT images in MM patients was proposed and implemented in a software tool for supporting visual assessment of MRD. The proposed segmentation evaluated the whole bone marrow, hindering the relationship between MFC results, taken from small and specific biopsy sites such as the iliac crest and sternum, and [^18^F]FDG PET visual assessment. For this reason, the main aim of this study is to estimate the representativeness of a single bone marrow biopsy in the evaluation of the whole bone marrow MRD. To this end, the bone marrow of the biopsy locations in [^18^F]FDG PET/CT images is segmented and quantified by radiomic features extraction. Then, these features are compared to whole bone marrow features and to MFC outcomes.

## Material and methods

### Subjects

Patients newly diagnosed with MM and treated at Hospital Universitario 12 de Octubre, Madrid, Spain, between 2013 and 2019 with assessment of MRD by both MFC and [^18^F]FDG PET–CT after the achievement of complete response are retrospectively included in the study cohort. Due to noise in the CT images or an incorrect position of the patient during acquisition, three patients are excluded, resulting in a study cohort of 39 cases. The study cohort is divided between PET+ and PET− based on the visual assessment of the [^18^F]FDG PET–CT by nuclear medicine experts. During the visual assessment, increased focal metabolic activity exclusive to the recent biopsy site is considered an inflammatory process and PET−. However, a significant imflammatory focal enhancement is usually not observed at the biopsy site, due to the small thickness of the needle used for the aspiration process and the time between tests. Patients are also grouped into MFC+ and MFC− according to MFC results. [^18^F]FDG PET/CT acquisition and MFC are performed based on the standards used by the Spanish Myeloma Group, as described in [9, 13], and were performed for each patient over a time period ranging from days to a maximum of two months between both evaluations.

### MFC acquisition and assessment

Bone marrow samples were collected from each patient to assess MRD by MFC when a complete response was reached. Erythrocyte-lysed whole bone marrow samples were immunophenotyped using a FACSCanto II flow cytometer (Becton–Dickinson, San Jose, CA) and analyzed by Infinicyt software (Cytognos, Salamanca, Spain), according to standards of the Spanish Myeloma Group. Samples in which aberrant immunophenotypic plasma cells were undetectable with a sensitivity between 10^–4^ and 10^–5^ were considered MRD−.

### Image acquisition

Siemens Biograph TruePoint 6 PET/CT (Siemens Healthineers, Erlangen, Germany) was used to obtain whole body [^18^F]FDG PET/CT scans. These images were acquired at the Department of Nuclear Medicine of the Hospital Universitario 12 de Octubre based on the European Association Nuclear Medicine (EANM) procedure guidelines [25]. An intravenous weight-adjusted shot of [^18^F]FDG with a mean dose of 352 ± 62.9 MBq was injected into the subjects. 50 to 60 min later, PET images were acquired with an emission time of 3 min per bed position. Random, scatter and attenuation corrections were performed. Reconstructed PET images have a matrix size of 168 × 168 with a voxel size of 4.0728 × 4.0728 × 5 mm^3^. Additionally, CT images were obtained using helical CTs (120–140 kVp, 25–170 mAs) with a resolution of 512 × 512 with a voxel size of 0.9766 × 0.9766 × 2.5 mm^3^.

### Image processing

The image preprocessing methodology and bone marrow segmentation is based on Milara et al. [24]. This segmentation is based on the application of different thresholding and morphological operations on the CT image to obtain de skeleton mask from the humeri, femora and torso regions. Then, spinal canal and compact bone are removed obtaining exclusively the bone marrow mask. All cases are visually reviewed and manually edited by an expert in Nuclear Medicine with the tool developed in Milara et al. [24]. Once this mask is obtained, a segmentation of the biopsy location is performed. Then, texture features and SUV metrics are extracted from the [^18^F]FDG PET image in the area characterized as biopsy on CT.

### Biopsy location segmentation

Per clinical practice in Hospital Universitario 12 de Octubre, three different biopsy sites are considered: sternum, left posterior iliac crest and right posterior iliac crest. The first step for each location is common, consisting on removing humeri and femora masks from the whole bone marrow mask. Then, each of the segmentation processes are developed separately.

For the sternum segmentation, the posterior half of the whole bone marrow mask, i.e., those voxels corresponding to the spine, are then removed. The same step is applied in the lower half of the torso mask in order to remove the pelvic region. Then, the longest component in the axial direction of the remaining mask is found and extracted, obtaining the sternum mask. Finally, only a cube of 40 voxels of edge length positioned at the upper location of the sternum remains in the final sternal mask, in order to more accurately represent the region of biopsy.

**Fig. 1:**
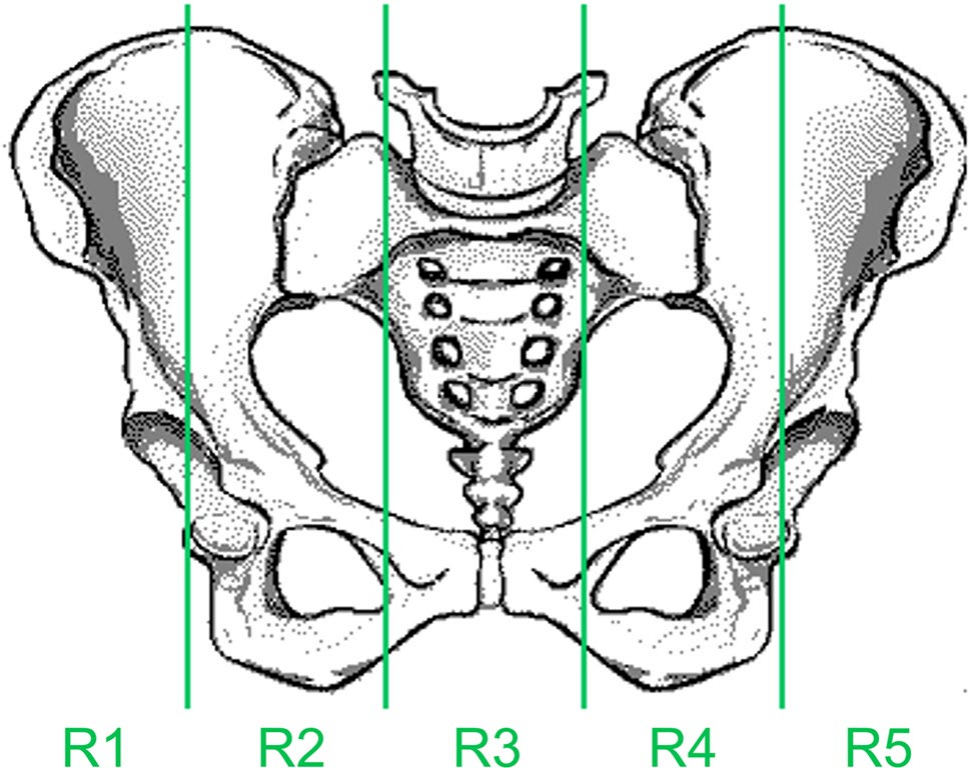
Illustration of the pelvic bone divided by four sagittal planes forming five distinct areas (R1–R5), with R1 being the region located on the far right and R5 the region located on the far left

Similarly to the sternum segmentation, for the posterior iliac crest segmentation, the superior half of the whole bone marrow mask is removed to eliminate the torso region. Then, the widest component in the sagittal plane is extracted to obtain solely the pelvis. The pelvis mask is divided into 5 regions (R1–R5) by 4 equidistant sagittal planes as shown in Fig. 1. In patients with the right posterior iliac crest as their biopsy location, only the first 2 (R1 and R2) out of these 5 regions remain on the mask. In those with left posterior iliac crest biopsy, only the last 2 (R4 and R5) are maintained.

**Fig. 2:**
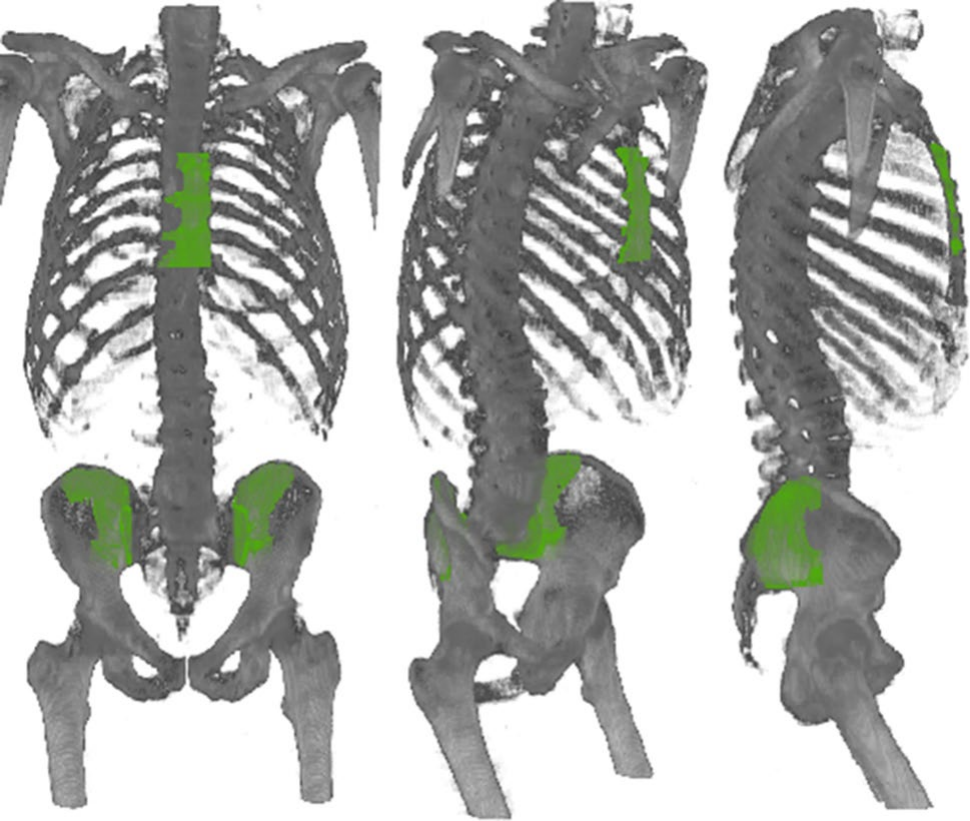
Segmentations of the bone marrow in biopsy locations (Sternum, Left Posterior Iliac Crest and Right Posterior Iliac Crest) represented in green overlays with the 3D reconstruction of a CT image

Finally, in both segmentations, the anterior iliac crest is removed by eliminating the anterior half of the remaining pelvis mask, obtaining exclusively posterior iliac crest. The final masks, represented over a 3D representation of the CT image in Fig. 2, are small enough to be representative for the biopsy, but also account for spatial variations of the specific puncture site for each patient. The predefined size, location, and shape for the three biopsy sites were reviewed and approved by experts in Nuclear Medicine.

For each patient, only the location of the biopsy obtained for the MFC assessment is segmented, other locations are discarded with the rest of the bone marrow mask. Subsequently, the texture features are extracted for biopsy location.

### Texture features extraction

Radiomics toolbox (https://github.com/mvallieres/radiomics, accessed on 12 September 2022) by M. Vallières for MATLAB [26–28] is used to quantized [^18^F]FDG PET images and to extract radiomic features. The quantization is performed using a uniform algorithm with 64 Gy levels, which scales the bone marrow mask intensity range linearly between 0 and the maximum SUVmax from the study cohort, following Eq. (1)

1 $$R\left( x \right) = round \left( {Ng \times \frac{I\left( x \right)}{{SUVmax}}} \right)$$

where *R(x)* is the rescaled value in voxel *x*, *I(x)* is the value in voxel *x* in the image before being rescaled and *Ng* the number of discrete gray level values. A total of 3 histogram-based features, 9 features from the grey-level co-occurrence matrix (GLCM), 13 features from the grey-level run length matrix (GLRLM), 5 features from Neighborhood Gray-Tone Difference Matrix (NGTDM) are extracted, along with the SUV_max_ and SUV_mean_.

### Statistical analysis and machine learning approach

Previous to the analysis, a Safe-Level-Synthetic Minority Over-Sampling Technique (Safe-Level-SMOTE) [29] is applied to the study cohort increasing the balance between PET+/PET− and MFC+/MFC− groups, obtaining an extra database. Every analysis is performed in both the original and oversampled databases for both pairs of groups.

Mann–Whitney U-test is applied to the texture features to assess differences between PET+/PET− and MFC+/MFC− groups. Moreover, the relationship between the feature magnitude and the respective class is estimated by Spearman’s rank correlation coefficients (ρ). For these analyses, SPSS Statistics Version 26.0 (IBM Corp., Armonk, NY) is used. Statistically significant differences are considered in analysis with a *p* value < 0.05. Over *p* value resulted, a multiple testing correction by means of Benjamini–Hochberg procedure is performed. Thus, a total of 4 Mann–Whitney-tests and 4 tests were performed to estimate Spearman correlation coefficients (to differentiate PET± and CBM±, both with the original database and with the oversampled database), performing for the 8 tests a correction by means of the Benjamini–Hochberg procedure. Finally, the correlation between the whole bone marrow mask and biopsy location mask quantification is tested in SPSS by analyzing the Spearman ρ of image features of both masks.

Orange 3.31 software (Bioinformatics Laboratory at the University of Ljubljana, Slovenia) is used for the application of eight ML classification algorithms [30] for PET+/PET− and MFC+/MFC− classification based on radiomic features and SUV metrics: decision tree, Support Vector Machine (SVM) with linear, polynomial and RBF kernels, random forest, logistic regression, k-nearest neighbors (kNN) and a neural network.

The decision tree is a sequential model that recursively organizes the information extracted from the training data into a hierarchical structure composed of nodes (attributes) and branches (classes) [31, 32]. Similarly, random forest is a model which combines many decision trees for prediction [33]. SVM is a classification algorithm that estimates the hyperplane equation that divides the input data into different output classes, maximizing the minimum distance between the classes and the hyperplane [34, 35]. Logistic regression is an algorithm by which a logistic curve is fitted to a training data set by modeling the probability of belonging to one of the classes. kNN is a machine learning model that stores training data and classifies new inputs as the class of the most k-nearest neighbors of the stored data. Finally, Neural networks are algorithms based on simple units called neurons or nodes. These nodes are connected to each other by simulating biological synapses and assigning weights to the connections that obtain better classifications [32].

The hyperparameters of the algorithms are defined in Supplementary material Table 1. For the internal validation of the model performance, a cross validation with five folds is used and six performance metrics are obtained: area under the curve (AUC) of the receiver operating characteristic curve, accuracy, F1-score (a weighted harmonic mean of precision and recall), precision (also known as positive predictive value), recall (or sensitivity) and specificity. For all these metrics, a value greater than 0.7 is considered acceptable, while a value greater than 0.9 is considered outstanding.

## Results

### Patients

Patient characteristics of the study cohort are shown in Table 1, which includes a total of 39 newly diagnosed with MM. Regarding PET visual assessment, 79.49% of patients are classified as PET−, whereas for biopsy evaluation 61.53% are MFC− cases. Additionally, sternum is observed as the preferred site for biopsy aspiration with a 56.41% followed by Left Iliac Crest with 38.46%. Discrepancies in 12 patients with PET−/MFC+ (38.71% of PET− cases) and 3 with PET+/MFC− (37.5% of PET+ cases) are observed.

**Table 1:** Demographics of the study cohort

	N	Sex (m/f)	Age (y ± SD)	MFC ( ±)	Biopsy location (S, LIC, RIC)
PET+	8	3/5	52.8 ± 8.5	3/5	5/3/2
PET−	31	8/23	57.6 ± 7.4	12/19	17/12/0
Total	39	11/28	56.6 ± 7.9	15/24	22/15/2

*S* sternum, *LIC* left iliac crest, *RIC* right iliac crest

### Radiomics analyses

Mann–Whitney U-tests results for the most significant texture features and SUV metrics for distinguishing between PET+ and PET− groups are shown in Table 2. For the original database, 19 out of 32 extracted characteristics are statistically significant, being only 8 of them significant after testing correction by means of Benjamini–Hochberg procedure. The lowest *p* values are obtained for SUV_max_ (*p *value = 0.002), followed by Entropy and GLN (Gray Level Non-Uniformity, *p *value = 0.007). On the other hand, for the oversampled database, most features (28 out of 32) show statistically significant differences between PET+ and PET− groups, even after testing correction by means of Benjamini–Hochberg procedure, obtaining 16 of them a *p* value < 0.05.

**Table 2:** Relationship between image features extracted from the bone marrow biopsy locations and PET classification including *p* value for Mann–Whitney U-test and ρ for Spearman correlation

Image feature	Original database			Oversampled		
	Mann Whitney (*p* value)	Spearman ρ	Spearman (*p* value)	Mann Whitney (*p* value)	Spearman ρ	Spearman (*p* value)
SUV_max_	0.002*	0.502**	0.001*	< 0.001*	0.662**	< 0.001*
SUV_mean_	0.237	0.192	0.242	0.014*	0.315**	0.013*
Energy	0.009*	− 0.423**	0.007*	< 0.001*	− 0.576**	< 0.001*
Contrast	0.028	0.355**	0.026	0.001*	0.444**	< 0.001*
Entropy	0.007*	0.434**	0.006*	< 0.001*	0.596**	< 0.001*
Homogeneity	0.015	− 0.395**	0.013*	< 0.001*	− 0.507**	< 0.001*
Correlation	0.040	0.333**	0.038	< 0.001*	0.470**	< 0.001*
SumAverage	0.065	0.299	0.064	0.005*	0.363**	0.004*
Variance	0.009*	0.423**	0.007*	< 0.001*	0.628**	< 0.001*
Dissimilarity	0.026	0.361	0.024	< 0.001*	0.459**	< 0.001*
AutoCorrelation	0.060	0.305	0.059	0.004*	0.370**	0.003*
SRE	0.010*	0.418**	0.008*	< 0.001*	0.551**	< 0.001*
LRE	0.031	− 0.350**	0.029	< 0.001*	− 0.506**	< 0.001*
GLN	0.007*	− 0.434**	0.006*	< 0.001*	− 0.610**	< 0.001*
RLN	0.012*	0.406**	0.010*	< 0.001*	0.547**	< 0.001*
RP	0.012*	0.406**	0.010*	< 0.001*	0.562**	< 0.001*
LGRE	0.076	− 0.288	0.076	0.004*	− 0.370**	0.003*
HGRE	0.047	0.322**	0.046	0.001*	0.415**	0.001*
SRLGE	0.154	0.389**	0.014*	0.028*	0.506**	< 0.001*
SRHGE	0.016	− 0.231	0.157	< 0.001*	− 0.282**	0.026*
LRLGE	0.040	− 0.333**	0.038	< 0.001*	− 0.452**	< 0.001*
RLV	0.028	− 0.355**	0.026	< 0.001*	− 0.455**	< 0.001*
Contrast (NGTDM)	0.251	0.186	0.256	0.006*	0.349**	0.005*
Complexity	0.024	0.367**	0.022	< 0.001*	0.511**	< 0.001*
Strength	0.237	0.192	0.242	0.018*	0.302**	0.017*
Variance (G)	0.465	0.118	0.473	0.025*	0.288**	0.023*
Skewness	0.028	0.355**	0.026	0.000*	0.448**	< 0.001*
Kurtosis	0.135	− 0.243	0.137	0.007*	− 0.343**	0.006*

The variables marked with * are significant after multiple testing correction by means of Benjamini–Hochberg procedure too. The variables marked with ** have a significative Spearman correlation at a 0.05 level (bilateral)

*SRE* short run emphasis, *LRE* long run emphasis, *GLN* gray level non-uniformity, *RLN* run length non-uniformity, *RP* run percentage, *LGRE* low gray level run emphasis, *HGRE* high gray level run emphasis, *SRLGE* short run high gray level emphasis, *SRHGE* short run high gray level emphasis, *LRLGE* long run low gray level emphasis, *RLV* run length variance

Spearman coefficients for the original database demonstrate positive correlations between PET+ cases and image features related to heterogeneity due to the heterogeneous pattern of the disease. This is the case for features like SUV_max_, Entropy, Variance, Short Run Emphasis (SRE), High Gray Level Run Emphasis (HGRE), Short Run High Gray Level Emphasis (SRLGE) and Complexity. Analyzing the resulting correlation coefficients, lower values of these variables are related to PET− cases, while higher values are with PET+. In contrast, features related to homogeneity like Energy, Gray Level Non-Uniformity (GLN), Low Gray Level Run Emphasis (LGRE), Long Run Low Gray Level Emphasis (LRLGE) and Run Length Variance (RLV) show negative correlation coefficients. For the oversampled database, almost all variables have a significant correlation, showing the same patterns.

Results from the Mann–Whitney U-tests showing statistically significant differences between MFC+ and MFC− patients are summarized in Table 3. Only 1 of the features is discriminative for MFC groups for the original database. Nevertheless, with the oversampled database, 8 of the features show statistically significant *p* values lower than SUV_max_, with the lowest being GLV (Gray Level Variance, *p* value = 0.004). None of the variables are significant after multiple testing correction by means of Benjamini–Hochberg procedure.

**Table 3:** Relationship between image features extracted from the bone marrow biopsy locations and MFC classification including *p* value for Mann–Whitney U-test and ρ for Spearman correlation

Image feature	Original database			Oversampled database		
	Mann Whitney (*p* value)	Spearman ρ	Spearman (*p* value)	Mann Whitney (*p* value)	Spearman ρ	Spearman (*p* value)
SumAverage	0.133	− 0.244	0.135	0.019	− 0.343**	0.017
AutoCorrelation	0.157	− 0.229	0.160	0.025	− 0.328**	0.023
LGRE	0.089	0.276	0.089	0.010	0.376**	0.008
HGRE	0.133	− 0.244	0.135	0.021	− 0.337**	0.019
SRLGE	0.166	− 0.220	0.178	0.017	− 0.319**	0.027
SRHGE	0.175	0.225	0.169	0.029	0.349**	0.015
LRLGE	0.126	0.248	0.128	0.021	0.337**	0.019
GLV	0.021	− 0.375**	0.019	0.004	− 0.415**	0.003

There are no variables significant after multiple testing correction by means of Benjamini–Hochberg procedure too. The variables marked with ** have a significative Spearman correlation at a 0.05 level (bilateral)

*GLV* gray level variance

PET+/PET− visual assessment by experts in Nuclear Medicine is done considering the whole bone marrow, not only the biopsy site. For this reason, a Spearman rank correlation coefficient (ρ) between image features of whole bone marrow mask and the same features for biopsy location mask are obtained. The statistically significant correlations are shown in Table 4. The highest correlation is observed for SRHGE (*ρ* = 0.853) and most of the extracted features are significantly correlated between the biopsy and the whole bone marrow analyses.

**Table 4:** Results from Spearman rank correlation analysis between images features of complete bone marrow and biopsy location

Image feature	Spearman	
	*ρ*	*p* value
SUV_max_	0.634	< 0.001
Energy	0.729	< 0.001
Contrast	0.709	< 0.001
Entropy	0.755	< 0.001
Homogeneity	0.773	< 0.001
SumAverage	0.693	< 0.001
Variance	0.570	< 0.001
Dissimilarity	0.750	< 0.001
AutoCorrelation	0.721	< 0.001
SRE	0.757	< 0.001
LRE	0.570	< 0.001
GLN	0.652	< 0.001
RLN	0.750	< 0.001
RP	0.680	< 0.001
LGRE	0.675	< 0.001
HGRE	0.762	< 0.001
SRHGE	0.853	< 0.001
LRLGE	0.740	< 0.001
GLV	0.554	< 0.001
RLV	0.495	< 0.001
Complexity	0.572	< 0.001

### Machine learning approach

Performance results for PET+/PET− classification by ML models based on all radiomic features are shown in Table 5 for the original database. None of them show an outstanding performance, as only acceptable values are obtained for 3 out of 6 performance metrics: AUC, accuracy and specificity. However, every model acquires a great specificity with values between 0.806 and 0.986.

**Table 5:** Classification performances of ML models with all image features extracted from the bone marrow biopsy locations for PET+ and PET− classification for the original database

Original database PET+/PET−						
Method	AUC	Accuracy	F1-score	Precision	Recall	Specificity
Decision tree	0.636	**0.769**	0.400	0.429	0.375	**0.871**
SVM-RBF	**0.743**	**0.769**	0.000	0.000	0.000	***0.968***
SVM-polynomial	**0.867**	**0.769**	0.308	0.400	0.250	***0.903***
SVM-linear	**0.838**	**0.718**	0.267	0.286	0.250	**0.839**
Random forest	**0.701**	**0.795**	0.333	0.500	0.250	***0.935***
Neural network	**0.793**	**0.718**	0.353	0.333	0.375	**0.806**
Logistic regression	**0.731**	**0.718**	0.353	0.333	0.375	**0.806**
kNN	0.563	**0.744**	0.000	0.000	0.000	***0.935***

Values in bold are considered acceptable (> 0.7). Values in italics are considered outstanding (> 0.9)

ML models based on radiomic features with significant differences between PET+/PET− groups in Mann–Whitney U-test are developed, obtaining results similar to those of the models based on all variables (Supplementary material Table 2). AUC values obtain better outcomes while specificity reach lower values.

On the other hand, performance metrics for PET+/PET− classification in the oversampled database based on all radiomic features is shown in Table 6. In this case, all ML models achieve acceptable performance with values > 0.7 in all metrics. The most outstanding performance is reached by Random Forest model, highlighting its great ability to distinguish PET+ cases. Nonetheless, SVM algorithm with RBF and Polynomial kernels also achieve remarkable results. In general, all models obtain AUC values between 0.786 and 0.974 and recall with values between 0.774 and 1.

**Table 6:** Classification performances of ML models with all image features extracted from the bone marrow biopsy locations for PET+ and PET− classification for the oversampled database

Oversampled database PET+/PET−						
Method	AUC	Accuracy	F1-score	Precision	Recall	Specificity
Decision tree	**0.786**	**0.790**	**0.794**	**0.781**	**0.806**	**0.774**
SVM-RBF	***0.960***	**0.871**	**0.875**	**0.848**	***0.903***	**0.839**
SVM-polynomial	***0.950***	**0.855**	**0.873**	**0.775**	***1.000***	**0.710**
SVM-linear	**0.856**	**0.823**	**0.836**	**0.778**	***0.903***	**0.742**
Random forest	***0.974***	**0.887**	**0.892**	**0.853**	***0.935***	**0.839**
Neural network	**0.879**	**0.823**	**0.831**	**0.794**	**0.871**	**0.774**
Logistic regression	**0.897**	**0.790**	**0.806**	**0.750**	**0.871**	**0.710**
kNN	**0.840**	**0.774**	**0.774**	**0.774**	**0.774**	**0.774**

Values in bold are considered acceptable (> 0.7). Values in italics are considered outstanding (> 0.9)

ML models for PET+/PET− classification based on radiomic features with *p *values < 0.05 after Mann–Whitney U-testing are also developed, obtaining results which are almost the same than those of the models based on all variables (Supplementary material Table 3). Moreover, the same ML models, based on all radiomic for original database (Supplementary material Table 4) and models based on all radiomic features and only on those with *p* values < 0.05 for oversampled database (Supplementary material Tables 5 and 6, respectively), are tested for the MFC+/MFC− classification. However, performance metrics of these models result in non-acceptable results.

## Discussion

Bone marrow biopsy/aspiration combined with [^18^F]FDG PET/CT images are the most common techniques to evaluate MRD, which has shown to correlate with survival outcomes. In this study, bone marrow of the biopsy locations from [^18^F]FDG PET/CT images of MM patients is segmented in order to develop a quantitative analysis by extracting radiomic features.

In the segmentation process, standardization of the regions to be extracted is needed since the exact puncture is unknown. For this reason, the proposed biopsy location segmentation contemplates a relatively wide region compared to the sample taken in the MFC technique.

Radiomic features extracted from [^18^F]FDG PET/CT allow to distinguish between PET+ and PET− cases. Specifically, 19 features (7 GLCM, 9 GLRLM, 1 NGTDM, 1 histogram-based feature and SUV_max_) for the original database and 28 features (9 GLCM, 11 GLRLM, 3 NGTDM, 3 histogram-based features, SUV_mean_ and SUV_max_) for the oversampled database out of 32 extracted features show significant differences. Indeed, 17 of them with *p* values < 0.001 for the oversampled database. Comparing the results with those obtained in the study of Milara et al. [24] where the whole bone marrow is evaluated, a greater representativeness of the MRD affectation in the biopsy regions than in the whole bone marrow can be observed, even though the visual analysis was performed for the whole body image.

The usefulness of radiomic features for heterogeneous pattern quantification is demonstrated by Spearman rank correlation coefficients (ρ), since the best variables in the prediction of PET+ cases are those with significant positive correlation coefficients, matching with heterogeneity-related variables such as Entropy or Variance. In contrast, homogeneous pattern is observed in PET− cases, since those features which represent homogeneity, such as Homogeneity and Energy, obtains significant negative coefficients. These results are in line with those of Milara et al. [24] since similar correlation values are obtained. As a result, biopsy locations are observed to be representative in the heterogeneous MRD pattern quantification.

Mann–Whitney U-test is unable to detect differences in radiomics features extracted from bone marrow biopsy locations between MFC+ and MFC− cases, similar to the case of using the complete bone marrow mask [24]. Taking into account the number of discrepancies in the [^18^F]FDG PET/CT visual assessment and MFC analyses with 12 patients with PET−/MFC+ and 3 with PET+/MFC− and the limited number of patients, the difficulty of differentiating MFC status by analyzing radiomics features extracted from the PET image was expected. Only 8 of the radiomic features show significant differences for the oversampled database before multiple testing correction by means of Benjamini–Hochberg procedure. Moreover, correlation analyses for image features and MFC result show a weak relationship between homogeneity-related features and MFC+ cases. These results could be a consequence of the non-representativeness of the biopsy, due to the sample taken or the sensitivity of the applied technique for the analysis, or the lack of accuracy in the extension or location of the bone marrow site segmented from the image, as well as the time difference between the biopsy and the [^18^F]FDG PET/CT image acquisitions, which could result in a progression of the patient towards CR between biopsy and image acquisition. For these reasons, two findings are observed: (1) both acquisitions, PET and MFC, are necessary for the MRD evaluation and (2) taking these acquisitions in a shorter period of time than two months may reduce the discrepancies found between PET+/MFC− and PET−/MFC+ cases. The order and time between acquisitions, and the specific percentage of immunophenotypically aberrant plasma cells in the MFC assessment, may result in different discrepancies in the assessments, which are caused by the recovery or relapse of the patient, or inflammatory processes due to the biopsy. For this reason, these factors should be reviewed in each individual case and will be taken into account in future works.

Spearman rank correlation analysis between radiomic features extracted from whole and biopsy location bone marrow results in 11 significant strong positive correlation (ρ > 0.7, *p* value < 0.05, bilateral). This comparison between the results of evaluating radiomic features based on whole body bone marrow [24] and bone marrow from biopsy site in the current study prove how the posterior iliac crest as well as the sternum are representative regions of the heterogeneous pattern of MRD commonly evaluated in the visual assessment of the whole bone marrow on [^18^F]FDG PET images.

ML results demonstrate the improvement of classification models when using a balanced database, comparing original and oversampled data measurements. Moreover, comparing models based on all radiomic features to those considered statistically significant in the Mann–Whitney U-test, the non-significant variables do not notably improve the classification results, since performance metrics show similar values. Performance of ML models applied in oversampled data suggest the possibility of developing models based on radiomic features as a Clinical Decision Support Systems (CDSS) for the PET+/PET− classification in MM patients, being the most remarkable Random Forest with every metric superior to 0.8 highlighting its AUC of 0.974 and SVM algorithms reaching acceptable values for every metric in all three kernels tested, with the RBF kernel obtaining the values closest to those of Random Forest. However, using biopsy site segmentation exclusively obtains similar performances to ML models based on whole bone marrow segmentation [24]. According to the results obtained in the Mann–Whitney U-tests and performance metrics, ML MFC+/MFC− classification models based on radiomic features are not feasible in our series. However, ML MFC+/MFC− classification models for the oversampled database focusing on biopsy sites generally obtain slightly better results (see Supplementary material Table 5), than models developed with the global bone marrow radiomic features (see Supplementary material Table 3 in Milara et al. [24]).

To the best of our knowledge, no other studies include bone marrow segmentation exclusively of the biopsy location in [^18^F]FDG PET/CT images from patients with MM at MRD assessment. Radiomics analysis has evolved and being used for diagnostic and prognostic prediction of multiple pathologies [17–19], but, until this study, only Milara et al. [24] have applied radiomic features for MRD assessment in MM patients. However, Han et al. [20] compared radiomic features of manually drawn volumes of interest to bone marrow biopsy results for diffuse large B cell lymphoma. The authors observed non-significant increases in biopsy positive cases for SUV metrics and two radiomic features (high grey-level zone emphasis and short-zone high grey-level emphasis) extracted from the iliac crest volumes of interest. Regarding the ML approach, the model performance in the classification of PET+/PET− for MRD assessment in our study obtain similar results to those obtained by Mesguich et al. [23] for the diagnosis of diffuse bone marrow infiltration in MM. In their study, Random Forest classifier also showed the best performance with a mean accuracy of 0.91 and AUC of 0.90 over 100 iterations (0.887 and 0.974 in our study, respectively). However, their study used only five radiomic features compared to 32 of our study.

Limitations of this study include a small and unbalanced cohort of patients (31 PET− and 8 PET+, 15 MFC+ and 24 MFC−). Despite the fact of having oversampled the database in order to balance the PET+/PET− and MFC+/MFC− groups, new created data is composed of original data and, therefore, information is still limited. On the other hand, due to the small and unknown specific location of the biopsy site inside the sternum or iliac crest, the segmentations were defined to include a slightly larger area while not encompassing the whole anatomical region. This limits the reproducibility of MFC+/MFC− analysis through [^18^F]FDG PET/CT image quantification. Nonetheless, it allows observing the representativeness of these regions in the MRD assessment. Considering discrepancies between PET and MFC assessments (12 with PET−/MFC+ and 3 PET+/MFC−), MFC analyses based on image features are limited by the time between both acquisitions, since the same state of progression of the disease is not being evaluated. Regarding the study of radiomics characteristics, only 32 features are extracted, while other studies include more than 2000. A future work with a wider variety of features is proposed to be developed. Lastly, the performance of the ML models could be improved using both hyperparameter optimization and different feature selection algorithms. Thus, both performance improvements along with the use of a wider variety of ML models is proposed as a future work.

Overall, the analyses proposed in this study lead to confirm the potential of radiomic features extracted from biopsy locations of [^18^F]FDG PET/CT images for the MRD assessment in patients diagnosed with MM. Furthermore, the representativeness of biopsy sites, iliac crest and sternum, in assessing the heterogeneous nature of the disease is demonstrated. Concerning ML results, future works lead to development of new models, based on hyperparameter optimization, capable of detecting patients with persistent MRD in bone marrow by quantification of [^18^F]FDG PET/CT images.

## Conclusions

In clinical routine practice, a combination of bone marrow biopsy and visual assessment of [^18^F]FDG PET/CT images are acquired for MRD evaluation in MM patients. However, results of both techniques are commonly inconsistent. In this study, an automatic segmentation methodology of the bone marrow at predefined biopsy sites is proposed. Radiomics analysis reveal significant differences in the metabolic uptake patterns at the biopsy sites with ML models accurately detecting PET+ patients.

## Supplementary Information

Below is the link to the electronic supplementary material.

Supplementary file1 (PDF 235 kb)
