# Supplementary material for: Radiomics analysis of bone marrow biopsy locations in [18F]FDG PET/CT images for measurable residual disease assessment in multiple myeloma
Source: Phys Eng Sci Med. 2023 May 8;46(2):903–13. doi: 10.1007/s13246-023-01265-0 (PMC10209284; doi:10.1007/s13246-023-01265-0)
Supplement: Supplementary file 1 — Supplementary file1 (PDF 235 kb) [file 13246_2023_1265_MOESM1_ESM.pdf]

Radiomics analysis of bone marrow biopsy locations in [^{18}F]FDG PET/CT images for measurable residual disease assessment in multiple myeloma

Authors: Eva Milara[1], Rafael Alonso[2,3,4,5], Lena Masseing[1], Alexander P. Seiffert[1], Adolfo Gómez-Grande[5,6], Enrique J. Gómez[1,7], Joaquín Martínez-López[2,3,4,5], Patricia Sánchez-González[1,7]

[1] Biomedical Engineering and Telemedicine Centre, ETSI Telecomunicación, Center for Biomedical Technology, Universidad Politécnica de Madrid, 28040 Madrid, Spain

[2] Department of Hematology and Instituto de Investigación Sanitaria (imas12), Hospital Universitario 12 de Octubre, 28041 Madrid, Spain

[3] Clinical Research Hematology Unit, Centro Nacional de Investigaciones Oncológicas (CNIO), 28029 Madrid, Spain

[4] Centro de Investigación Biomédica en Red Cáncer (CIBERONC), Madrid, Spain

[5] Facultad de Medicina, Universidad Complutense de Madrid, 28040 Madrid, Spain

[6] Department of Nuclear Medicine, Hospital Universitario 12 de Octubre, 28041 Madrid, Spain

[7] Centro de Investigación Biomédica en Red de Bioingeniería, Biomateriales y Nanomedicina, Instituto de Salud Carlos III, 28029 Madrid, Spain

Corresponding author:

P. Sánchez-González (p.sanchez@upm.es)

Supplementary material

Table 1. Selected values for hyperparameters of the machine learning models.

**Method**	**Hyperparameters**
Decision Tree	Minimum of 2 instances per leave; subsets greater or equal to 2 instances; maximal tree depth of 100; classification when majority reaches 95%.
SVM	Cost (C): 1; regression loss epsilon (ε): 0.1; tolerance: 0.001; Max. iterations: 100<br>For Polynomial kernel with equation $(g\ x \cdot y + c)^d$: g=auto, c=1, d=3.
Random Forest	Number of trees: 20; subsets greater or equal to 2 instances;
Neural Network	Neurons in hidden layers: 50; Activation: Identity; Solver: Adam; regularization α= 0.8; Max. iterations: 150; with replicable training.
Logistic Regression	Regularization Lasso; Strength = 1.
kNN	Number of neighbors: 2; metric: Euclidean, wright: uniform.

Table 2. Classification performances of ML models based on image features extracted from the bone marrow biopsy locations with a *p*-value<0.05 after Mann-Whitney U-testing for PET+ and PET- classification for the original database. Values in bold are considered acceptable (>0.7). Values marked with * are considered outstanding (>0.9).

Original database PET+/PET-						
**Method**	**AUC**	**Accuracy**	**F1-score**	**Precision**	**Recall**	**Specificity**
Decision Tree	0.677	**0.769**	0.471	0.444	0.500	**0.839**
SVM-RBF	**0.883**	**0.795**	0.200	0.500	0.125	***0.968***
SVM-Polynomial	***0.917***	**0.846**	0.625	0.625	0.625	***0.903***
SVM-Linear	***0.921***	**0.744**	0.375	0.375	0.375	**0.839**
Random Forest	**0.813**	**0.795**	0.500	0.500	0.500	**0.871**
Neural Network	***0.905***	**0.795**	0.556	0.500	0.625	**0.839**
Logistic Regression	**0.800**	**0.769**	0.400	0.429	0.375	**0.871**
kNN	0.638	0.692	0.000	0.000	0.000	**0.871**

Table 3. Classification performances of ML models based on image features extracted from the bone marrow biopsy locations with a *p*-value<0.05 after Mann-Whitney U-testing for PET+ and PET- classification for the oversampled database. Values in bold are considered acceptable (>0.7). Values marked with * are considered outstanding (>0.9).

Original database PET+/ PET-						
**Method**	**AUC**	**Accuracy**	**F1-score**	**Precision**	**Recall**	**Specificity**
Decision Tree	**0.786**	**0.790**	**0.794**	**0.781**	**0.806**	**0.774**
SVM-RBF	***0.956***	**0.887**	**0.889**	**0.875**	***0.903***	**0.871**
SVM-Polynomial	***0.965***	**0.855**	**0.873**	**0.775**	**1.000**	**0.710**
SVM-Linear	**0.893**	**0.806**	**0.818**	**0.771**	**0.871**	**0.742**
Random Forest	***0.956***	**0.871**	**0.875**	**0.848**	***0.903***	**0.839**
Neural Network	**0.895**	**0.823**	**0.831**	**0.794**	**0.871**	**0.774**
Logistic Regression	***0.922***	**0.823**	**0.831**	**0.794**	**0.871**	**0.774**
kNN	**0.837**	**0.742**	**0.724**	**0.778**	0.677	**0.806**

Table 4. Classification performances of ML models with all image features extracted from the bone marrow biopsy locations for MFC+ and MFC- classification for the original database. Values in bold are considered acceptable (>0.7). Values marked with * are considered outstanding (>0.9).

Original database MFC+/ MFC-						
**Method**	**AUC**	**Accuracy**	**F1-score**	**Precision**	**Recall**	**Specificity**
Decision Tree	0,392	0,436	0,313	0,294	0,333	0,500
SVM-RBF	0,410	0,513	0,174	0,250	0,133	**0,750**
SVM-Polynomial	0,360	0,590	0,429	0,462	0,400	**0,708**
SVM-Linear	0,373	0,667	0,606	0,556	0,667	0,667
Random Forest	0,530	0,538	0,308	0,364	0,267	**0,708**
Neural Network	0,653	0,615	0,545	0,500	0,600	0,625
Logistic Regression	**0,707**	0,615	0,483	0,500	0,467	**0,708**
kNN	0,397	0,564	0,000	0,000	0,000	***0,917***

Table 5. Classification performances of ML models with all image features extracted from the bone marrow biopsy locations for MFC+ and MFC- classification for the oversampled database. Values in bold are considered acceptable (>0.7). Values marked with * are considered outstanding (>0.9).

Oversampled database MFC+/ MFC-						
**Method**	**AUC**	**Accuracy**	**F1-score**	**Precision**	**Recall**	**Specificity**
Decision Tree	0,567	0,583	0,600	0,577	0,625	0,542
SVM-RBF	0,482	0,625	0,625	0,625	0,625	0,625
SVM-Polynomial	0,554	0,563	0,588	0,556	0,625	0,500
SVM-Linear	0,594	0,646	0,667	0,630	0,708	0,583
Random Forest	0,626	0,563	0,588	0,556	0,625	0,500
Neural Network	0,664	0,729	0,745	0,704	0,792	0,667
Logistic Regression	0,706	0,688	0,717	0,655	0,792	0,583
kNN	0,607	0,625	0,500	0,750	0,375	0,875

Table 6. Classification performances of ML models based on image features extracted from the bone marrow biopsy locations with a *p*-value<0.05 after Mann-Whitney U-testing for MFC+ and MFC- classification for the oversampled database. Values in bold are considered acceptable (>0.7). Values marked with * are considered outstanding (>0.9).

Oversampled database MFC+/ MFC-						
**Method**	**AUC**	**Accuracy**	**F1-score**	**Precision**	**Recall**	**Specificity**
Decision Tree	0.533	0.542	0.542	0.542	0.542	0.542
SVM-RBF	0.442	0.521	0.465	0.526	0.417	0.625
SVM-Polynomial	**0.717**	0.521	0.549	0.519	0.583	0.458
SVM-Linear	0.688	0.625	0.609	0.636	0.583	0.667
Random Forest	0.646	0.625	0.609	0.636	0.583	0.667
Neural Network	**0.717**	0.583	0.565	0.591	0.542	0.625
Logistic Regression	0.608	0.604	0.596	0.609	0.583	0.625
kNN	0.604	0.625	0.526	**0.714**	0.417	**0.833**
